# Artificial Intelligence—What to Expect From Machine Learning and Deep Learning in Hernia Surgery

**DOI:** 10.3389/jaws.2024.13059

**Published:** 2024-09-06

**Authors:** Robert Vogel, Björn Mück

**Affiliations:** Klinikum Kempten - Klinikverbund Allgäu, Kempten, Germany

**Keywords:** AI, machine learning, deep learning, hernia surgery, neural network

## Abstract

This mini-review explores the integration of Artificial Intelligence (AI) within hernia surgery, highlighting the role of Machine Learning (ML) and Deep Learning (DL). The term AI incorporates various technologies including ML, Neural Networks (NN), and DL. Classical ML algorithms depend on structured, labeled data for predictions, requiring significant human oversight. In contrast, DL, a subset of ML, generally leverages unlabeled, raw data such as images and videos to autonomously identify patterns and make intricate deductions. This process is enabled by neural networks used in DL, where hidden layers between the input and output capture complex data patterns. These layers’ configuration and weighting are pivotal in developing effective models for various applications, such as image and speech recognition, natural language processing, and more specifically, surgical procedures and outcomes in hernia surgery. Significant advancements have been achieved with DL models in surgical settings, particularly in predicting the complexity of abdominal wall reconstruction (AWR) and other postoperative outcomes, which are elaborated in detail within the context of this mini-review. The review method involved analyzing relevant literature from databases such as PubMed and Google Scholar, focusing on studies related to preoperative planning, intraoperative techniques, and postoperative management within hernia surgery. Only recent, peer-reviewed publications in English that directly relate to the topic were included, highlighting the latest advancements in the field to depict potential benefits and current limitations of AI technologies in hernia surgery, advocating for further research and application in this evolving field.

## Introduction

“A computer would deserve to be called intelligent if it could deceive a human into believing that it was human” [1] a phrase once coined by Alan Mathison Turing, who is widely regarded as a pioneer in computer science and artificial intelligence. Turing’s early substantial contributions to AI in the mid-20th century, notably his role in deciphering the Enigma code during World War II, laid the foundation for the field [2].

**FIGURE 1:**
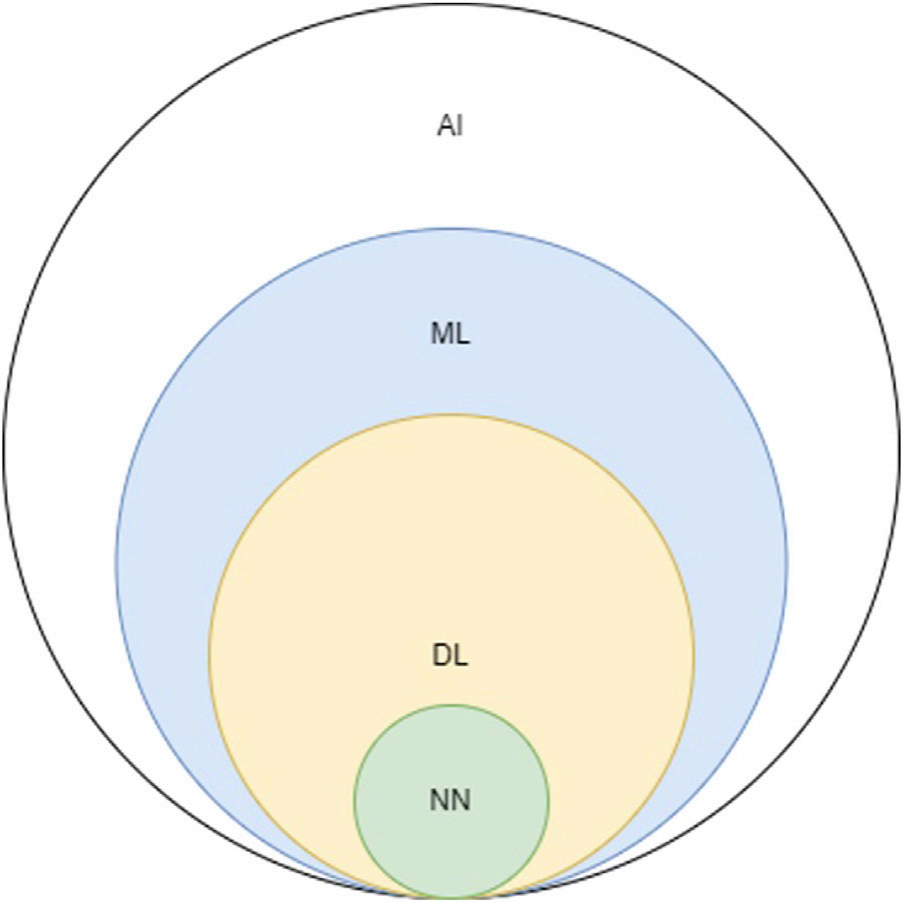
Schematic depicting the relationship between AI, artificial intelligence; ML, machine learning; DL, deep learning; NN, neural networks.

Adopting Turing’s quote to contemporary times, one could identify any device capable of producing realistic videos, photos, and conversations as intelligent since artificial Intelligence (AI) has reached a level of sophistication where it can convincingly mimic human behavior. As a result, the term AI is now used ubiquitously as it is entangled within a vast area in modern computer science. Consequently, related terms are often incorrectly used interchangeably [3]. For simplicity reasons, AI can be considered an umbrella term that includes concepts such as machine learning (ML), neural networks (NN), and deep learning (DL) (Figure 1).

Many of these technologies have naturally also found applications in medicine, including hernia surgery. Machine learning algorithms (MLA) for example can, in simple terms, learn from existing data, generalize them, and extrapolate future, yet unknown data, allowing them to perform tasks without explicit instructions [4]. For instance, Hassan et al [5] trained an MLA on preoperative clinical data to accurately predict complications in abdominal wall reconstruction (AWR) surgery.

Classical machine learning algorithms typically rely on structured, labeled data (i.e., data organized according to a set hierarchy) to make predictions, making them generally more dependent on human intervention for learning. In contrast, deep learning (DL), being a subset of machine learning, does not necessarily require labeled datasets. Deep Learning algorithms can be regarded both as a refined as well as mathematically complex evolution of machine learning algorithms. To put it illustratively, just as a human brain uses its neural pathways to process information and reach decisions, deep learning employs artificial neural networks that simulate this process. These networks are capable of learning from data in an incremental manner, which enables them to make complex deductions as more information becomes available. It can utilize raw data, such as text, images, and videos, to identify features and distinguish explicit patterns, thus discovering data groupings without human interference [6–8].

**FIGURE 2:**
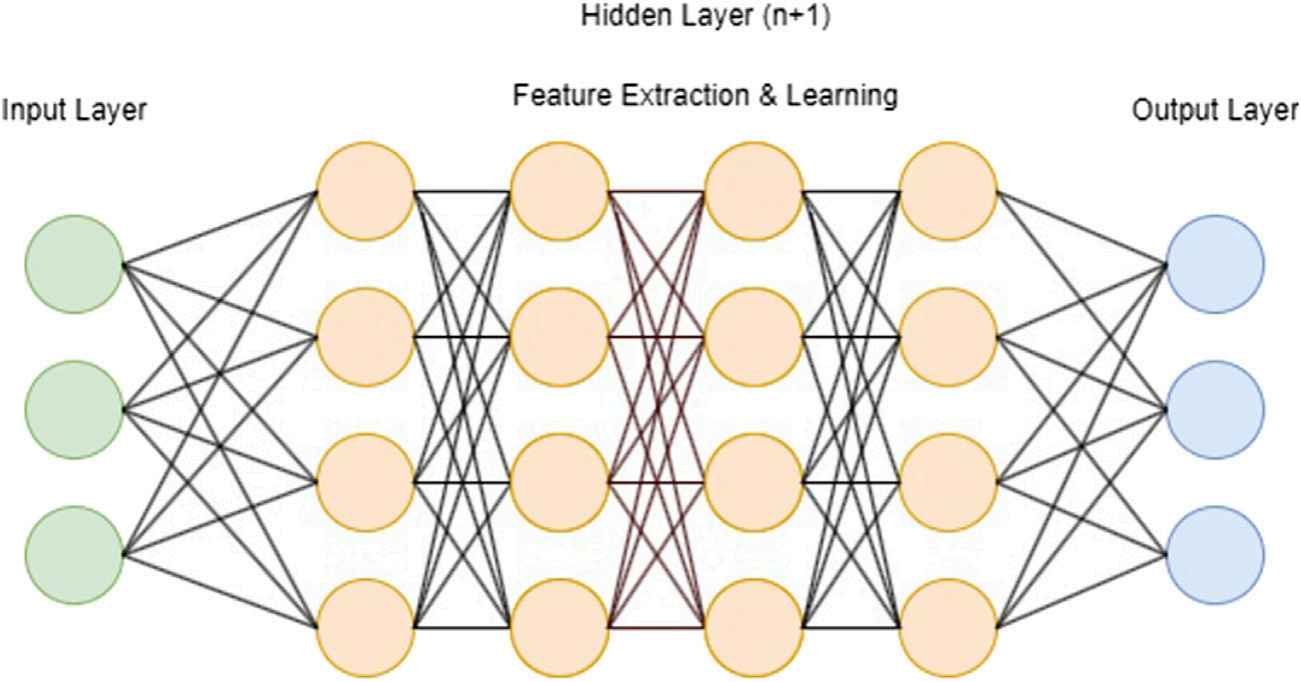
Schematic visualization of a deep learning model with input layer, n + 1 hidden layers and output layer).

The distinction between ML and DL is defined by the number of layers in a neural network also known as hidden layers (Figure 2) [9]. If more than three of these layers are present, the algorithm is considered a DL model. In summary, hidden layers act as intermediary stages between the input and output of a neural network. They play a crucial role in capturing complex patterns in data, which makes neural networks highly effective for diverse applications such as image and speech recognition and natural language processing. The configuration and weighting of these hidden layers are essential for creating effective neural network models. In particular, DLMs (deep learning models) involved in computer-aided diagnosis have been successfully applied in cranial [10, 11] trauma [12, 13] and oncologic [14–16] computed tomography (CT) analysis. In this context, Elhage et al. [17] have demonstrated that an 8-layered convolutional neural network (CNN), a type of DL architecture, can effectively predict surgical complexity in AWR procedures, competing with a panel of expert surgeons. Furthermore, DLMs have proven to be effective in predicting surgical outcomes and post-operative complications [18–20].

The aim of this mini-review is to explore the advancements and applications of AI, particularly ML and DL, as well as other notable advancements in computing science in the field of hernia surgery, highlighting their potential benefits and current limitations.

## Methodology

In conducting this mini review, we utilized literature from several databases including, PubMed and Google Scholar. The key terms used in our search were: “hernia,” “artificial intelligence, “deep learning,” “machine learning,” “abdominal wall reconstruction,” “hernia surgery,” and “augmented reality.”

Inclusion criteria for this review involved publications directly relevant to the study topic. Specifically, we included studies that focused on preoperative planning, intraoperative imaging and techniques, as well as postoperative management and follow-up in the context of abdominal wall reconstruction. We considered articles published in peer-reviewed journals, studies published in English, and publications from the last 10 years to ensure the inclusion of the most recent advancements.

Publications were excluded if they were not related to hernia surgery, studies not involving AI, ML, DL, or augmented reality in a surgical context, and non-peer-reviewed articles such as opinion pieces, editorials, or non-scientific reports. We also excluded articles focusing on animals or *in vitro* models rather than human subjects, and duplicate studies or publications with overlapping data sets.

## Discussion

The potential impact of computing sciences on medicine was already being anticipated in the 1970s. W B Schwartz [21] predicted that “computing science would likely have significant effects by enhancing and possibly even replacing certain intellectual functions traditionally performed by physicians.” The author noted that the integration of computers in medical practice could profoundly influence physician manpower and the quality of healthcare. Schwartz’s foresight highlighted the transformative potential of computing science in medicine. 52 years later in 2022, a protocol was developed by Saeidi et al. [22] that enables a robot to autonomously perform a small bowel anastomosis with minimal human interference.

The rapid advancements in AI hence raise the question of not whether AI will shape our surgical future, but rather how it will do so.

In a quality improvement study conducted by Elhage et al. [17], the researchers aimed to assess the potential of image-based deep learning in predicting the complexity of AWR surgeries, specifically the need for component separation, as well as predicting pulmonary and wound complications. To achieve this, they developed an 8-layer CNN capable of analysing image characteristics.

The study design involved a comparison of the CNN-based surgical complexity model with a validation set of CT-images. The validation set was evaluated by a panel of 6 expert AWR surgeons, who were blinded to the surgical complexity DLM. The dataset utilised for analysis comprised 369 patients and 9303 CT images.

In summary, Elhage et al. found that the CNN-based DLM was more accurate than expert surgeon judgment in predicting the surgical complexity of AWR procedures [81.3% compared to the surgeons’ accuracy of 65.0% (p < 0.001)]. Furthermore, they observed that an additional DLM accurately predicted the occurrence of surgical site infections [AUC 0.898 (p < 0.001)]. These findings highlight the potential of image-based DLMs as valuable tools in forecasting surgical outcomes and improving decision-making in AWR surgery.

In another article published by Hassan et al. [5], the authors displayed the effectiveness of machine learning models (MLMs) in predicting hernia recurrence (HR), surgical site occurrences (SSOs), and 30-day readmission. Their study reported that MLMs achieved mean accuracy rates of 85% (95% CI 80%–90%) for HR prediction, 72% (95% CI 64%–80%) for SSOs, and 84% (95% CI 77%–90%) for 30-day readmission. These ML algorithms, trained on readily available preoperative clinical data, proved to be highly accurate in forecasting complications associated with AWR-surgery. The authors concluded to support the integration of MLMs into the preoperative evaluation process for patients undergoing AWR.

To facilitate the advancements of AI in hernia surgery, it is in our view crucial to expand patient databases on an international scale. Increasing the diversity and size of these databases will provide a broader range of data for training AI models and allow for more comprehensive and accurate predictions.

Since the effectiveness of AI predictions relies heavily on both the accuracy and the thoroughness of the input data, access to diverse patient populations will help address potential biases and ensure the reliability of AI algorithms across different demographics [23]. Expanding patient databases internationally can also help identify patterns and trends that may not be apparent in smaller or more localised datasets.

Moreover, the collection and analysis of video data in surgical procedures hold great potential for advancing the field of hernia surgery. By capturing surgical procedures through video recordings, it becomes possible to create detailed anatomical maps and explore the integration of AR with robotics in surgical interventions [24]. By integrating DLMs on video data, it appears to be possible to extract relevant information, such as anatomical landmarks, tissue characteristics, and procedural phase recognition, which can contribute to the development of more precise surgical interventions [25]. Additionally, the combination of video data with augmented reality has the potential to enhance surgical visualisation and navigation as well as robotic surgery training-simulators. By possibly overlaying real-time anatomical information and guidance onto the surgeon’s view, AR could provide valuable assistance during complex hernia surgeries. Surgeons can benefit from visual cues, real-time feedback, and enhanced precision, ultimately leading to improved surgical outcomes. For example, Cui et al. [26] used a CNN model comprised out of surgical videos from 35 patients in laparoscopic hernia repair in order to detect the vas deferens and their results suggested that the CNN promptly identifies and visualises vas deferens images.

Another promising approach combines wide-field, planar, near-infrared fluorescence imaging with AI for automated real-time guidance during surgery as highlighted by Gioux et al. [27]. This technology could help identify and avoid hidden tissues, such as nerves or blood vessels covered by fatty or connective tissue, by highlighting their location within the surgical field. Like following a breadcrumb trail, the AI system could guide surgeons in the direction of dissection, enhancing precision and hence reducing the risk of complications.

An essential pillar of AI is characterized through the concept of phase recognition. This involves the utilization of MLAs and computer vision techniques to automatically discern and categorize distinct phases or stages within a process.

Phase recognition AI utilizes algorithms, frequently rooted in DL, to scrutinize patterns, features, and temporal sequences within video data. This capability allows the system to differentiate between diverse stages of the surgical process [28, 29].

In a recently published study by Takeuchi et al. [30], the primary objective was to develop a DL-based automated phase-recognition system for identifying surgical phases in Transabdominal Preperitoneal (TAPP) procedures (i.e. preparation, peritoneal flap incision, peritoneal flap dissection, hernia dissection, mesh deployment, mesh fixation, peritoneal flap closure, and additional closure). A secondary aim was to explore the correlation between surgical skills and the duration of each phase. An AI model (AIM) was trained to automatically recognize surgical phases from videos, and the study assessed the relationship between phase duration and surgical skills. A fourfold cross-validation was used to evaluate the AIMs performance, achieving accuracies of 88.81% and 85.82% for unilateral and bilateral cases, respectively.

Ortenzi et al. [31] reported on an AI-based computer vision algorithm designed to automatically recognize surgical steps in videos of totally extraperitoneal (TEP) inguinal hernia repair. The algorithm achieved an overall accuracy of 88.8% in recognizing the complete procedure. The per-step accuracy was highest for the hernia sac reduction step at 94.3% and lowest for the preperitoneal dissection step at 72.2%. The authors concluded that this novel AIM could provide fully automated video analysis with a high level of accuracy. High-accuracy AIMs that enable automation of surgical video analysis allow for the identification and evaluation of surgical performance.

However, the integration of video data and DLMs in hernia surgery research and practice requires the accumulation of diverse and high-quality video datasets. It seems obvious, that these datasets should encompass various surgical techniques, patient characteristics, and procedural variations to ensure the robustness and generalisability of AIMs [32]. To optimize this process, Hashimoto et al. [33] propose extensive collaborations among surgeons as well as data scientists. These efforts are crucial to facilitate the sharing and pooling of video data, which will accelerate the development and refinement of AI-driven approaches in hernia surgery.

In a scoping review published by Taha et al. [34], the authors provided a comprehensive summary of the current objectives related to the integration of AI in the field of hernia surgery. They highlighted the potential applications and benefits of AI in areas such as medical imaging and surgical training. However, the authors also acknowledged the limited number of publications available on this specific topic, indicating a gap in the existing literature.

Based on this observation, Taha et al. emphasised the need for further research and the publication of original articles to explore and investigate the ways in which AI can effectively assist in medical imaging and support the training of surgeons in the context of hernia surgery.

Furthermore, ever since AI has emerged, ethical considerations have played a significant role in discussions surrounding the technology. While the potential benefits are undeniable, questions arise regarding accountability if complications occur. O’Sullivan et al. [35] categorise responsibility into accountability, liability, and culpability. Drawing a parallel to self-driving cars, they compare the surgeon overseeing the hypothetical autonomous robot to the driver of a car, making the surgeon ultimately responsible for the robot’s actions. Undeniably, a robust legal framework is paramount prior to implementing semi-autonomous programs or machines into any medical field.

In conclusion, further research on the applications of AI is undoubtedly of crucial importance. Moreover, we believe that promoting international collaboration in expanding patient and video databases are indispensable in facilitating this process.
